# Preflight Spectral Calibration of Airborne Shortwave Infrared Hyperspectral Imager with Water Vapor Absorption Characteristics

**DOI:** 10.3390/s19102259

**Published:** 2019-05-16

**Authors:** Honglin Liu, Dong Zhang, Yueming Wang

**Affiliations:** 1 Key Laboratory of Space Active Opto-Electronics Technology, Shanghai Institute of Technical Physics of CAS, Shanghai 200083, China; 15921212812@163.com (H.L.); 15518289839@163.com (D.Z.); 2 University of Chinese Academy of Sciences, Beijing 100049, China

**Keywords:** hyperspectral imager, short wave infrared, laboratory spectral calibration, semiconductor lasers, water vapor absorption spectrum

## Abstract

Due to the strong absorption of water vapor at wavelengths of 1350–1420 nm and 1820–1940 nm, under normal atmospheric conditions, the actual digital number (DN) response curve of a hyperspectral imager deviates from the Gaussian shape, which leads to a decrease in the calibration accuracy of an instrument’s spectral response functions (SRF). The higher the calibration uncertainty of SRF, the worse the retrieval accuracy of the spectral characteristics of the targets. In this paper, an improved spectral calibration method based on a monochromator and the spectral absorptive characteristics of water vapor in the laboratory is presented. The water vapor spectral calibration method (WVSCM) uses the difference function to calculate the intrinsic DN response functions of the spectral channels located in the absorptive wavelength range of water vapor and corrects the wavelength offset of the monochromator via the least-square procedure to achieve spectral calibration throughout the full spectral responsive range of the hyper-spectrometer. The absolute spectral calibration uncertainty is ±0.125 nm. We validated the effectiveness of the WVSCM with two tunable semiconductor lasers, and the spectral wavelength positions calibrated by lasers and the WVSCM showed a good degree of consistency.

## 1. Introduction

As an image–spectrum merging technology, hyperspectral imaging has been widely used in agriculture, ocean observations, urban planning, disaster monitoring, and many other fields [1,2,3]. The quantitative retrieval of a target’s surface spectral reflection characteristics is one of the important features of hyperspectral imagers, requiring an accurate spectral position for the instrument. For an imaging spectrometer with a 10 nm full width at half maximum (FWHM), a spectral shift of 1 nm shows relative errors of up to ±25% in the measured radiance near strong atmospheric absorption valleys [4]. The Jet Propulsion Laboratory (LaKan Yada and Pasadena, America) has reported that a measured radiance error of about 8% occurs when spectral calibration accuracy approaches 5% of the FWHM in the atmospheric absorption range [5]. High-precision spectral calibration is indispensable in the field of hyperspectral remote sensing applications. The popular methods for spectral calibration are divided into two main categories: The characteristic spectrum calibration method (CSCM), which relies on sources with unique spectral properties, such as tunable lasers [6], filters containing rare earth oxides [7], atmospheric characteristic absorption lines [8,9], gas molecules absorb cells [10], spectrum lamps [11], etc. The other is a monochromatic and collimator-based wavelength scanning calibration method, called the monochromatic collimation light calibration method (MCLCM). The advantage of CSCM is that it is easy to operate and can quickly detect the spectral offset of the spectrometers. Its disadvantage is that the spectral characteristic is untunable or the tunable spectrum range is narrow and cannot cover the whole operation spectral range of the spectrometer. The absolute uncertainty of CSCM can approach 10% of the spectrum sampling interval of the responsive channel, which could meet practical requirements.

The monochromatic collimation light calibration method can realize high-precision continuous wavelength scanning in a wide spectral range, which is generally adopted by scholars. One of moderate resolution imaging spectroradiometer’s (MODIS) on-orbit calibration methods is to use a spectro-radiometric calibration assembly (SRCA) to calibrate the offset of center wavelength and the deviation of FWHM [12,13]. Zadnik et al. successfully calibrated the spectral response function of compact airborn spectral sensor (CAMPASS) in a laboratory using a high-resolution monochromator with an absolute calibration accuracy of ±0.5 nm [14]. The monochromatic collimated spectrum calibration method has gradually become the first choice for spectral calibration of spectrometers, but the uncertainty of the monochromator’s stability has always been the bottleneck limiting the accuracy of spectrometers. In response to such problems, Zhang et al. [15] analyzed the relationship between the mechanical error of the monochromator system and the wavelength of the emitted light and established a mathematical model to calculate the monochromatic light’s wavelength offset. The calibration accuracy is ±0.3  nm. The European Space Agency calibrated the monochromator via a HeNe laser and a series of gas atomic lamps (Hg, Ne, Ar, Kr, and Xe). The absolute spectral uncertainty of the airborne prism experiment (APEX) was increased to ±0.15 nm [16]. With the enhancement of the spectral resolution of the hyperspectral imager, the calibration accuracy of MCLCM in laboratory is continuously improving. However, for hyperspectral imagers with a 3-nm spectral sampling interval, the effect of water vapor absorption on the spectral calibration accuracy of the channels located in an absorptive wavelength range is gradually being understood. Unlike the hyperspectral imagers in a space remote sensing field, laboratory spectral calibration for hyperspectral imagers in an aerial remote sensing application was performed under a normal atmospheric environment. Due to the strong absorptive effect of water vapor in 1350–1420 nm and 1820–1940 nm, the actual digital number (DN) response curves of hyperspectral imagers obtained by MCLCM deviate from the Gaussian shape, which leads to a decrease in the calibration accuracy of the channel’s spectral response function. We studied the actual DN response curve in the wavelength range mentioned above and found that each absorption valley along the actual DN response curve of every spectral channel located in the absorptive range corresponds to the spectral absorption characteristics of water vapor. This phenomenon not only helps us to solve the problem of the decrease in the spectral calibration accuracy of the spectral channels, but also corrects the wavelength offset of the monochromator simultaneously. The water vapor spectral calibration method (WVSCM) is an improved laboratory spectral calibration method based on monochromator and transmittance characteristics of water vapor. WVSCM can promote the application of hyperspectral imagers in the aerospace field.

In the second section of this paper, we introduce the laboratory spectral calibration principle and the water vapor spectrum calibration method for the hyperspectral imagers. In Section 3, the experimental verification of the WVSCM and the wavelength offset of the monochromator being removed simultaneously are described. The Section 4 provides the error analysis and effectiveness validity of the WVSCM. The experimental results in this paper were consistent with the theory, which confirmed the feasibility of the water vapor spectrum calibration method.

## 2. Methods

The spectral response function of an imaging spectrometer applies the relative response ability of each spectral channel to different wavelength monochromatic light. The spectral response function can be expressed as a convolution of the slit function, the spectrometer optical system line spread function, and the detector pixel response function [17]. The hyper-spectrometer uses an optical system of a prism or grating to split light and map between objects and their images. A charge-coupled device (CCD) focal plane detector and its electronic system are used to accomplish the process of photoelectric information conversion, amplification, sampling, quantization, and coding. If we assume the spectral radiance at a monochromator’s exit slit is *L*(*λ*), the atmospheric transmittance is *v*(*λ*), the energy transfer efficiency of the optical system is *τ*(*λ*), the distribution function of the dispersive system is *ψ*(*λ*), the quantum efficiency of the CCD pixels is *η_d_*(*λ*), the spectral response efficiency of the electronic system is *η_e_*(*λ*), the charges’ conversion coefficient from the quantum well to CCD output voltage is *κ*, the total voltage gain coefficient of the detector’s matching circuits is *G*, the total noise voltage introduced by the process of photoelectric conversion and voltage amplification is *n*, the quantization bit of the ADC chip is *m*, and the quantization reference voltage is *V_REF_*, then the DN value generated during the integral time *T* can be expressed as

(1) $$DN=\frac{\left(2^{m}-1\right)}{V_{REF}}\cdot G\cdot \kappa \cdot \frac{\pi \cdot {\left(d\cdot D\right)}^{2}}{4\cdot f^{2}\cdot h\cdot c}\cdot \int_{0}^{T}\int_{\lambda 1}^{\lambda 2}L\left(\lambda \right)\cdot v\left(\lambda \right)\cdot \tau \left(\lambda \right)\cdot \psi \left(\lambda \right)\cdot \eta_{e}\left(\lambda \right)\cdot \eta_{d}\left(\lambda \right)\cdot \lambda d\lambda dt+\frac{\left(2^{m}-1\right)}{V_{REF}}\cdot n$$

where d is the size of the detector’s pixel, D is the aperture of the optical system, f is the focal length of optical system, and h and c are the Planck constants and the speed of light, respectively. If we use k and b to represent the absolute radiation transfer coefficient of the hyperspectral imager, and S(λ) as the spectral response function, then Equation (1) can be rewritten as

(2) $$DN=k\cdot \int_{\lambda 1}^{\lambda 2}L\left(\lambda \right)\cdot v\left(\lambda \right)\cdot S\left(\lambda \right)d\lambda +b$$

For practical convenience, the spectral response function S(λ) is usually expressed by a Gaussian function [18], as shown in Equation (3). The subscripts *i* and *j* in Equation (3) denote the pixel in the *j-*th spatial sequence and the *i*-th spectrum sequence of the CCD focal plane. $\lambda_{c}$ is the center wavelength position, FWHM is the full width at half maximum, and A is the relative spectral response efficiency of pixel *i j*:

(3) $$f_{i,j}\left(\lambda \right)=A\cdot \exp\left(-\frac{{\left(\lambda -\lambda_{c}\right)}^{2}}{{\left(FWHM/2\sqrt{ln2}\right)}^{2}}\right)$$

If the wavelength of a bundle of monochromatic light received by the spectrometer is recorded as $\lambda_{0}$, the DN response value of pixel *i j* can be written as

(4) $$DN=k\cdot L\left(\lambda_{0}\right)\cdot v\left(\lambda_{0}\right)\cdot S\left(\lambda_{0}\right)+b$$

In the wavelength range without the effect of atmospheric absorption, the DN value is the linear gain of the spectral response function. It can be accurately calibrated by the actual DN response curve. However, when the influence of the atmosphere cannot be ignored, especially in the strong absorptive wavelength range of 1350 nm to 1420 nm and 1820 nm to 1940 nm caused by water vapor, the actual DN response curve deviates from the Gaussian shape, which cannot reflect the characteristic of the spectral response function accurately. Take pixel *i j* located within the strong absorptive range as an example, then we use Gaussian function ${Func}_{i,j}^{Ori}$*(λ)* shown in Equation (5) to represent its original DN response function, which is obtained by Gauss fitting of the pixel’s actual DN curve: ${DN}_{i,j}^{prac}$(*λ*):

(5) $$Func_{i,j}^{Ori}\left(\lambda \right)=P^{\prime }\cdot \exp\left(-\frac{{\left(\lambda -\lambda_{c}^{\prime }\right)}^{2}}{{\left(FWHM^{\prime }/2\sqrt{ln2}\right)}^{2}}\right)$$

${Func}_{i,j}^{Ori}$*(λ)* is just an approximation of ${Func}_{i,j}^{r}$(*λ*), which is the intrinsic DN response function of pixel *i j*. We can solve function ${Func}_{i,j}^{r}$(*λ*) by fitting the actual and the simulated (theoretical) DN response curve based on the spectral absorption characteristics of water vapor. If we set the atmospheric transmittance as *v*(*λ)* and introduce the relative DN response height variation ∆*P*, the offset of the center wavelength ∆*λ*, and the stretch of the full width at half maximum ∆*FWHM* into Equation (5), then we create the difference function E(ΔP,Δλ,ΔFWHM) to represent the difference distance between the simulated DN response curve and the actual one, which is expressed in Equation (6):

(6) $$E=||\left(P^{\prime }+\Delta P\right)\cdot \exp\left(-\frac{{\left[\lambda -\left(\lambda_{c}^{\prime }+\Delta \lambda \right)\right]}^{2}}{{\left(FWHM^{\prime }\cdot ∆FWHM/2\sqrt{ln2}\right)}^{2}}\right)\cdot v\left(\lambda \right)-DN_{i,j}^{prac}\left(\lambda \right){\left|\right|}_{2}^{2}$$

**Figure 1:**
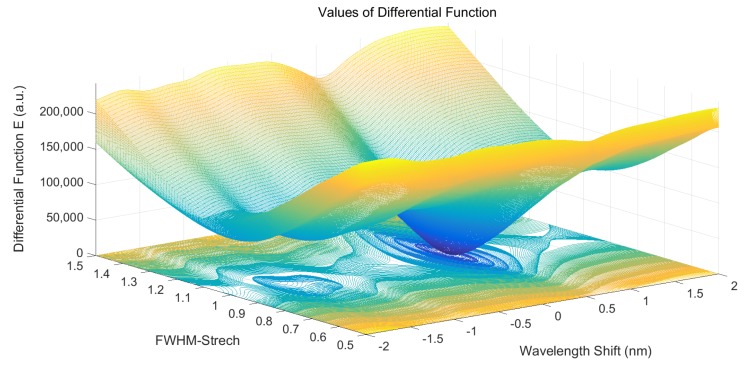
Solutions of the difference function E. The wavelength shift axis represents the central wavelength offset. The full width at half maximum (FWHM) stretch axis represents the deviation of the full width at half maximum. The vertical axis represents the values of the difference function E of different solutions.

The set of solutions in Equation (6) is a three-dimensional (3D) cube matrix, which is shown in Figure 1. By bringing the solutions of the global minimum value $E_{min}$ back into Equation (5), we can determine the real intrinsic DN response function ${Func}_{i,j}^{r}$*(λ)*, as shown in Equation (7). The parameter *P*_0_, *FWHM_stretch_*, and *λ_shift_* are the solutions of $E_{min}$:

(7) $$Func_{i,j}^{r}\left(\lambda \right)=\left(P^{\prime }+P_{0}\right)\cdot \exp\left(-\frac{{\left[\lambda -\left(\lambda_{c}^{\prime }+\lambda_{shift}\right)\right]}^{2}}{{\left(FWHM^{\prime }\cdot FWHM_{stretch}/2\sqrt{ln2}\right)}^{2}}\right)$$

The content mentioned above provides the theoretical basis and calculation process of the water vapor spectral calibration method (WVSCM). By repeatedly using the WVSCM, we could calibrate the intrinsic DN response functions of all spectral channels located in the wavelength range of 1350 nm to 1420 nm and 1820 nm to 1940 nm one at a time. The spectral response functions can be determined simultaneously. We verified the WVSCM through experiments, which are described in Section 3.

## 3. Experiment Validation

**Figure 2:**
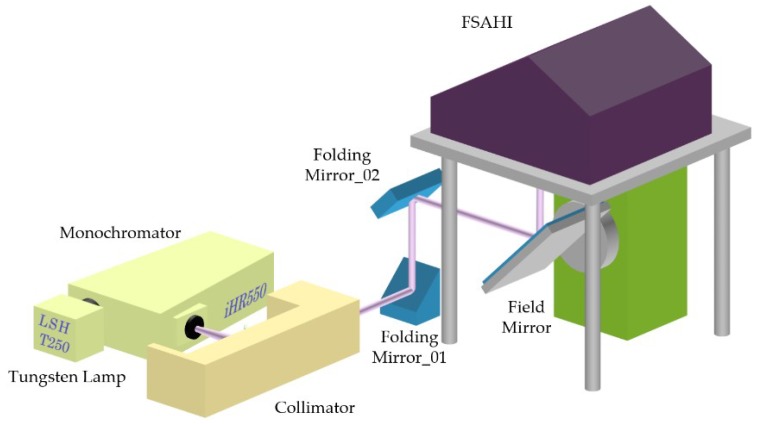
Laboratory spectral calibration structure of a full spectral airborne hyperspectral imager (FSAHI). LSH-T250 is a tungsten halogen source produced by HORIBA, Ltd., with a spectral coverage of 350 nm to 2400 nm, and iHR550 is a monochromator produced by HORIBA, Ltd.

The laboratory temperature was 18 degree Celsius, the relative humidity was 34%, and the partial pressure of water vapor in the air was around 700 Pascal. The optical distance of monochromatic light was about 3 m. The laboratory spectral calibration structure is shown in Figure 2.

The instrument for wavelength scanning was an iHR550 monochromator produced by HORIBA, Ltd. (Kyoto, Japan), and the calibration light source was an LSH-250 tungsten halogen lamp. The spectrometer for spectral calibration was a full spectral airborne hyperspectral imager (FSAHI) [19,20,21], which was developed by the Shanghai Institute of Technical Physics, Chinese Academy of Sciences (Shanghai, China). The main FSAHI parameters are shown in Table 1.

**Figure 3:**
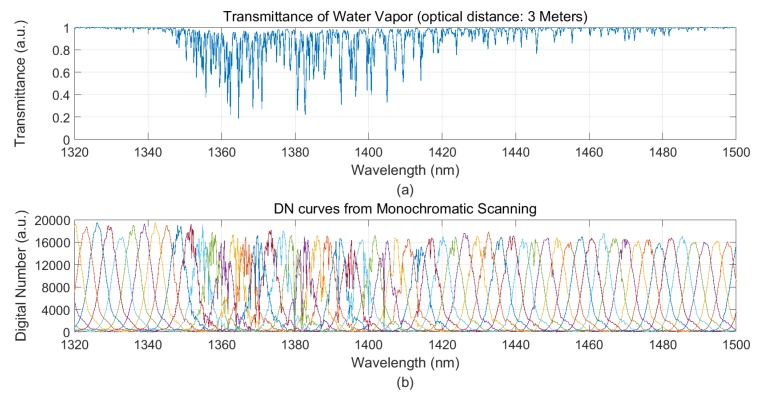
(**a**) Spectral transmittance characteristics of water vapor provided by MODTRAN (moderate resolution atmospheric transmittance and radiance code is a 2 cm^−1^ resolution band-model code, developed jointly by Spectral Sciences, Inc. and the Air Force Research Laboratory/Space Vehicles Directorate) in the wavelength range of 1320 nm to 1500 nm, and (**b**) the actual digital number (DN) response curves obtained by FSAHI.

**Figure 4:**
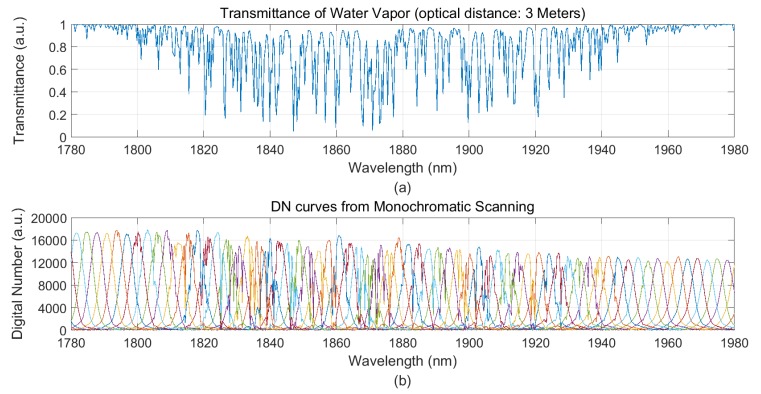
(**a**) Spectral transmittance characteristics of water vapor provided by MODTRAN in the wavelength range of 1780 nm to 1980 nm, and (**b**) the actual DN response curve obtained by FSAHI.

We selected the middle field view of FSAHI to receive the monochromatic light, and the wavelength scanning step of the monochromator was 0.2 nm. The actual DN response curves covering the range of 1320 nm to 1550 nm and 1780 nm to 1980 nm are shown in Figure 3 and Figure 4, respectively. The absorption of water vapor could cause the actual DN response curve to deviate from the Gaussian shape. This phenomenon is consistent with the theory presented in Section 2. 

**Figure 5:**
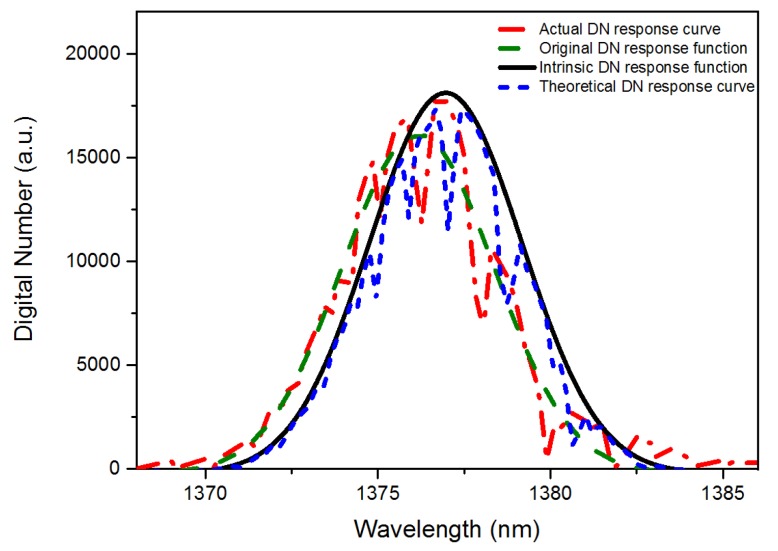
The curve set of the pixel’s DN response values and the calculation results of the water vapor spectral calibration method (WVSCM).

Taking the *i*-th channel with a center wavelength around 1376 nm in *j-*th spatial sequence as an example, we used the WVSCM to calculate the pixel’s intrinsic DN response function ${Func}_{i,j}^{r}$(*λ*), as shown by the solid black line in Figure 5. The red dotted and dashed line is the actual DN response curve ${DN}_{i,j}^{prac}$(*λ*). The green dashed line is the original DN response function curve obtained by Gaussian fitting of ${DN}_{i,j}^{prac}$(*λ*). The blue short dashed line in Figure 5 is the simulated DN response curve, which is the product of ${Func}_{i,j}^{r}$(*λ*) and *v*(*λ*) in the wavelength direction.

**Figure 6:**
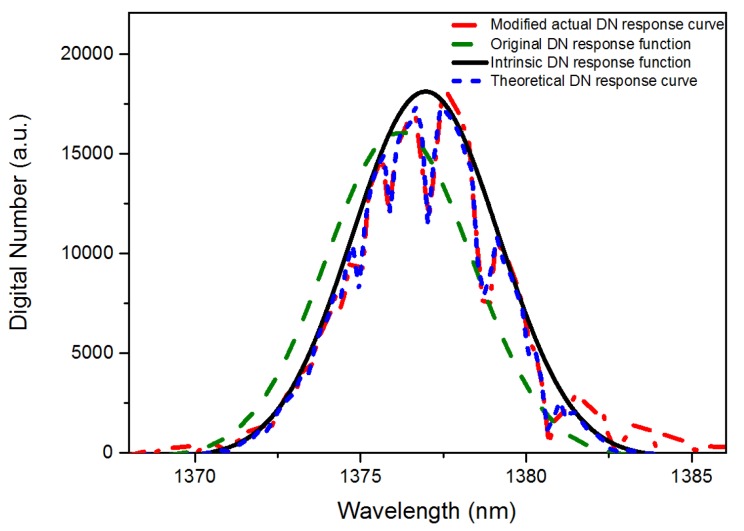
The corrected curve set of the pixel’s DN response values and the calculation results of the WVSCM.

Although, the simulated DN response curve has a high degree of similarity with the actual curve, a misalignment between them was apparent. This phenomenon is mainly caused by the difference between the spectral positions of the monochromator and the MODTRAN model. Since the state of monochromator changes with time, it is necessary to calibrate the spectral deviation of the monochromator using MODTRAN. We used the least-square method to fit ${DN}_{i,j}^{prac}$(*λ*) and the simulated response curve to obtain the best matching spectral position via translating the actual DN response curve, as shown in Figure 6. The translation distance of the actual DN response curve is the spectral offset of the monochromator, which we recorded as ∆*λ*_mono_.

**Figure 7:**
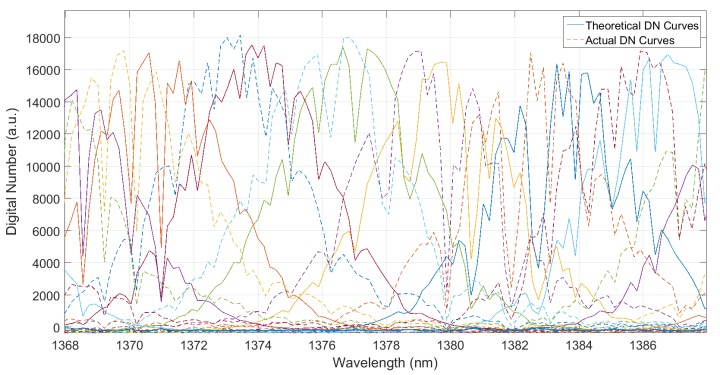
Comparison of the spectral positions of the actual and simulated DN response curves.

**Figure 8:**
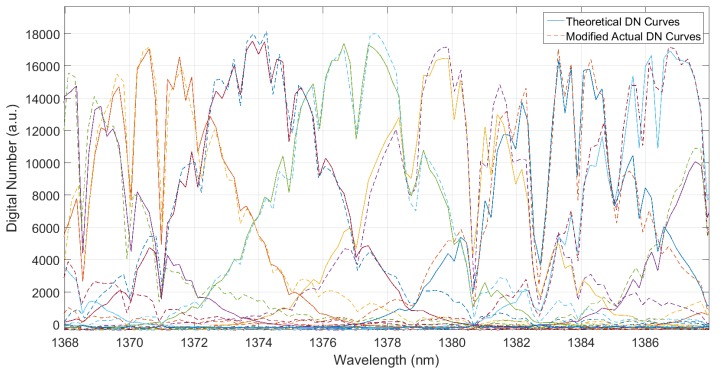
Comparison of the theoretical and actual DN response curves after spectral correction.

By using the WVSCM to analyze the channels of the *j-*th spatial sequence located in the wavelength range of water vapor absorption, the actual DN response curves and the simulated ones could be obtained, as shown in Figure 7. After completing the overall translation on the actual DN response curves with ∆*λ*_mono_, shown in Figure 8, the simulated and actual response curves were consistent with each other. The wavelength offset of the monochromator was removed.

## 4. Result Analysis

**Figure 9:**
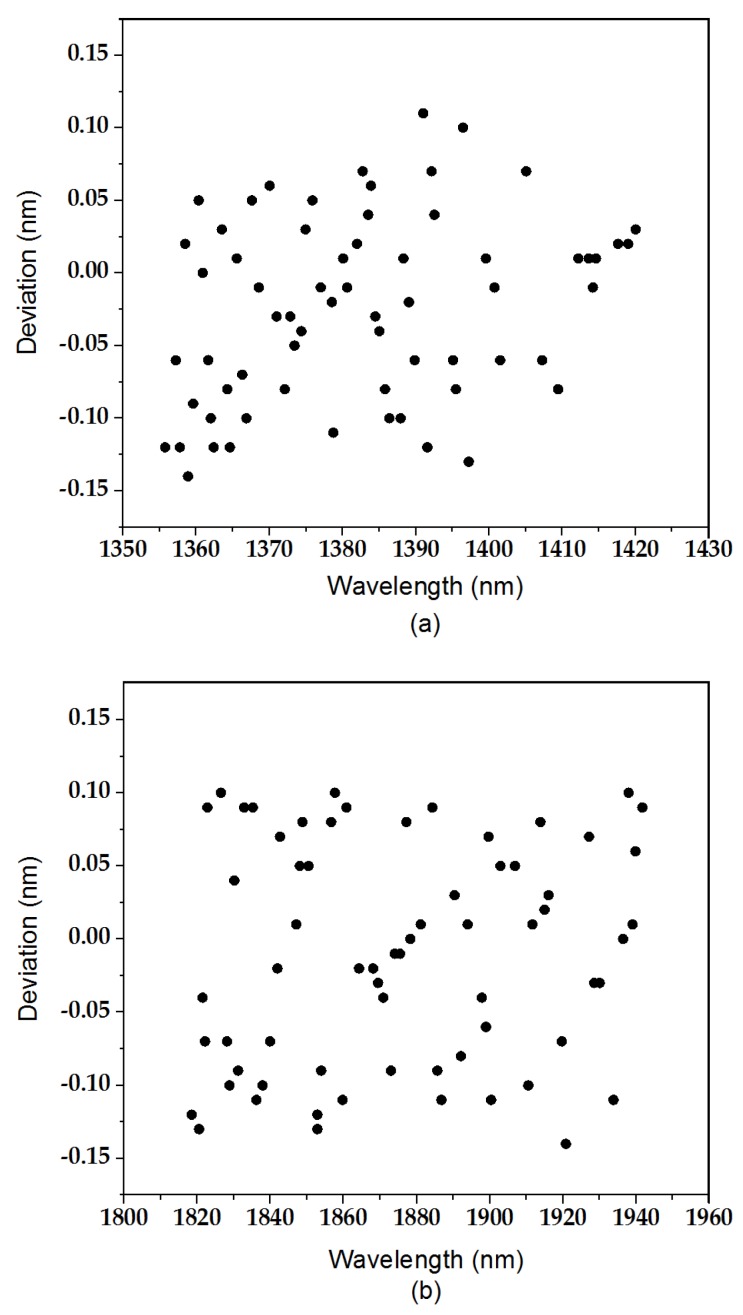
(**a**) Wavelength position offsets of absorptive valleys between ${\mathrm{DN}}_{i,j}^{prac}\left(\lambda \right)$ and simulated DN response curves in the wavelength range of 1350 to 1420 nm, and (**b**) wavelength position offsets of absorptive valleys between ${\mathrm{DN}}_{i,j}^{prac}\left(\lambda \right)$ and simulated DN response curves in the wavelength range of 1820 nm to 1940 nm.

According to the wavelength position of each absorptive valley of water vapor in the wavelength range of 1350 to 1430 nm and 1800 to 1960 nm provided by the MODTRAN model, we performed statistics on the wavelength position deviations of the 132 absorptive valleys between the translated actual DN response curves and the theoretical DN response curves. The wavelength deviations of each valley are shown in Figure 9.

Among the absorptive sample points, the maximum positive offset was 0.11 nm, and the maximum negative offset was 0.14 nm. Their root mean square error was 0.07 nm. The average full width at half maximum (FWHM) of FSAHI is 4.70 nm. Therefore, it is reasonable to think that the absolute accuracy of spectral calibration is ±0.125 nm. A 4.5% level of FWHM accuracy, which refers to three times the root mean square error, was achieved.

**Figure 10:**
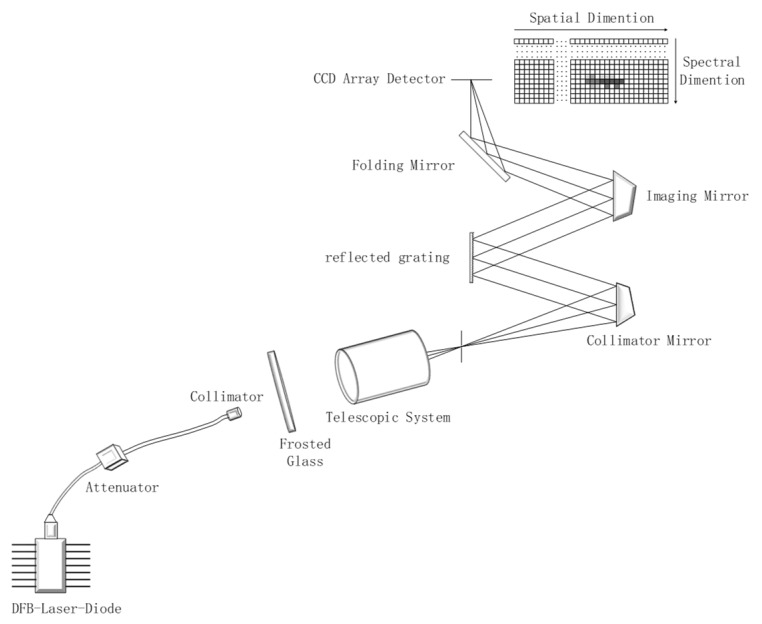
The optical structure of the hyperspectral imager and the spectral calibration structure with a single-frequency semiconductor laser (SFSL).

The WVSCM takes the spectral position of MODTRAN as the benchmark to calibrate the spectrometer and monochromator. To verify the effectiveness of MODTRAN, we performed an imaging experiment with two tunable single-frequency semiconductor lasers to examine the spectral calibration results of the WVSCM. The principle of the experiment is shown in Figure 10.

**Figure 11:**
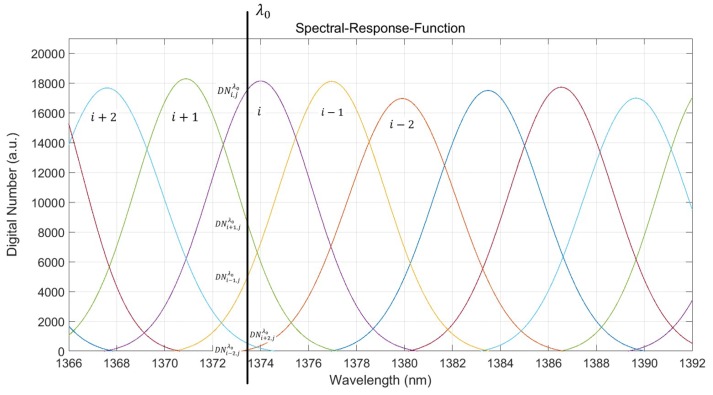
The spectral calibration principle of single-frequency semiconductor lasers.

The single-frequency semiconductor laser (SFSL) applied in this experiment used a Distributed Feedback (DFB) laser with an integrated Thermoelectric Cooler (TEC) module. By adjusting the magnitude of the drive current, small range modulation of the monochromatic light’s wavelength can be achieved [22,23]. Since the response efficiency of different spectral channels to the same monochromatic light differ, we ascertained the wavelength of the laser by a hyperspectral imager according to this phenomenon. We used the WVSCM to calibrate the intrinsic DN response functions of the *j-*th spatial sequence, as shown in Figure 11. The responsive DN values of the spectral channels that responded significantly to the monochromatic light *λ*_0_ arranged from large to small were recorded as: ${\mathrm{DN}}_{i,j}\left(\lambda_{0}\right)$, ${\mathrm{DN}}_{i+1,j}\left(\lambda_{0}\right)$, ${\mathrm{DN}}_{i-1,j}\left(\lambda_{0}\right)$, ${\mathrm{DN}}_{i+2,j}\left(\lambda_{0}\right)$ and ${\mathrm{DN}}_{i-2,j}\left(\lambda_{0}\right)$. Therefore, we defined the single-frequency spectral scaling loss function DELT(*λ*) in Equation (8), according to Figure 11:

(8) $$DELT\left(\lambda \right)=\sqrt{\overset{i+2}{\underset{k=i-2}{\sum }}[\gamma_{k,j}\cdot P_{k,j}\cdot \exp\left(-\frac{{\left(\lambda -\lambda_{c}^{k,j}\right)}^{2}}{{\left(FWHM_{k,j}/2\sqrt{ln2}\right)}^{2}}\right)-\gamma_{k,j}\cdot \frac{\left(DN_{k,j}\left(\lambda_{0}\right)-DN_{min,j}\left(\lambda_{0}\right)\right)}{\left(DN_{i,j}\left(\lambda_{0}\right)-DN_{min,j}\left(\lambda_{0}\right)\right)}{]}^{2}}$$

where $\gamma_{k,j}$ is the normalization gain coefficient of each channel’s intrinsic DN response function. The purpose of normalization is to eliminate the effects of noise levels in different channels. $DN_{min,j}$ is the minimum DN response value among the channels and $\rho_{k,j}$ is the matching weight of the channels, which decreases with decreasing $DN_{k,j}$.

**Figure 12:**
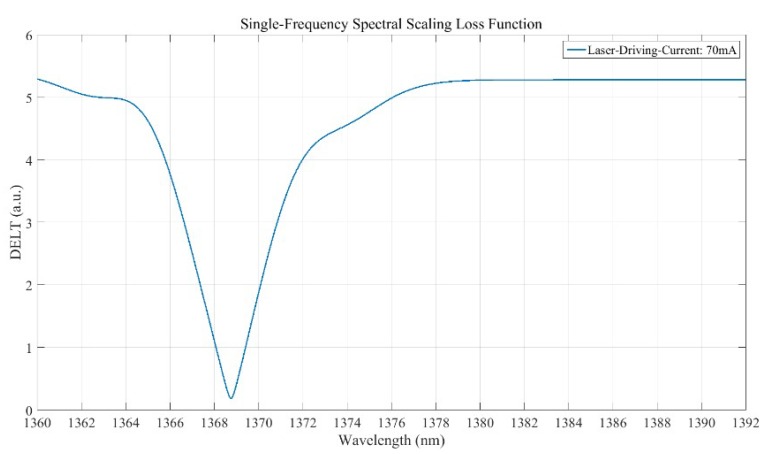
The calculation result of DELT(*λ*) when *λ*_0_ is 1368.813 nm.

The intrinsic wavelengths of lasers were calibrated using a HighFinesse-WS8 wavelength meter produced by the HighFinesse Company (Munich, Germany). The results of the loss function *DELT*(*λ*) are a function of wavelength *λ*, as shown in Figure 12. The wavelength position corresponding to the minimum value of DELT(*λ*) is the monochromatic wavelength of laser calibrated by FSAHI. The measurement results of two different semiconductor lasers measured by HifghFinesse-WS8 and FSAHI under different driving currents are shown in Table 2 and Table 3.

## 5. Conclusions

In this paper, we proposed a spectral calibration method based on water vapor transmission characteristics, which we named the water vapor spectrum calibration method (WVSCM). The method does not rely on the use of lasers or a series of gas atomic lamps to calibrate the monochromator beforehand, and it can not only remove the distortions of spectral channels in the wavelength range of 1350 nm to 1420 nm and 1820 nm to 1940 nm, which is caused by water vapor, but the decrese in laboratory spectral calibration accuracy can be reduced simultaneously. WVSCM is an economical and less time consuming laboratory spectral calibration method. The absolute spectral uncertainty of the method is ±0.125 nm, and the root mean square error is 0.07 nm. We used two tunable semiconductor lasers to verify the effectiveness of the water vapor spectrum calibration method. The calibration results of semiconductor lasers and the method are in accordance with each other. The WVSCM provides a new reference method for laboratory spectral calibration, and is helpful to promote the application of hyperspectral imagers.

## Figures and Tables

**Table 1:** Main full spectral airborne hyperspectral imager (FSAHI) parameters.

Index	Parameter
Operation Mode	Short Wave Infrared
Wavelength Coverage	1000–2500 nm
Spectral Channel Number	512
Instantaneous Field of View	0.5 mrad
Field of View	15°
Spectral Sampling Interval	3 nm

**Table 2:** Wavelength calibration results of tunable semiconductor laser 01.

Driving Current (mA)	Intrinsic Wavelength of Laser (nm)	Wavelength Calibrated by FSAHI (nm)	Deviation (nm)
70	1368.813	1368.742	0.068
80	1368.929	1368.854	0.074
90	1369.050	1369.005	0.045
100	1369.185	1369.241	−0.059
110	1369.326	1369.408	−0.078
120	1369.475	1369.439	0.040
130	1369.625	1369.716	−0.089

**Table 3:** Wavelength calibration results of tunable semiconductor laser 02.

Laser Power (dBm)	Intrinsic Wavelength of Laser (nm)	Wavelength Calibrated by FSAHI (nm)	Deviation (nm)
0.0	1550.270	1550.285	−0.010
1.1	1550.278	1550.292	−0.012
2.0	1550.286	1550.247	0.045
3.1	1550.300	1550.228	0.079
4.2	1550.319	1550.275	0.049
5.6	1550.358	1550.366	−0.002
7.2	1550.431	1550.452	−0.019
8.2	1550.508	1550.603	−0.092
9.2	1550.611	1550.600	0.011
9.9	1550.726	1550.649	0.085
10	1550.806	1550.940	−0.134
